# Amplexicaule A exerts anti-tumor effects by inducing apoptosis in human breast cancer

**DOI:** 10.18632/oncotarget.7848

**Published:** 2016-03-02

**Authors:** Meixian Xiang, Hanwen Su, Guangwen Shu, Dingrong Wan, Feng He, Morgann Loaec, Yali Ding, Jun Li, Sinisa Dovat, Gaungzhong Yang, Chunhua Song

**Affiliations:** ^1^ College of Pharmacy, South-Central University for Nationalities, Wuhan, PR China; ^2^ Pennsylvania State University College of Medicine, Department of Pediatrics, Hershey, PA, USA; ^3^ Renmin Hospital of Wuhan University, Wuhan, PR China

**Keywords:** amplexicaule A, apoptosis, Akt/mTOR, MCL-1, breast cancer

## Abstract

Chemotherapy is the main treatment for patients with breast cancer metastases, but natural alternatives have been receiving attention for their potential as novel anti-tumor reagents. Amplexicaule A (APA) is a flavonoid glucoside isolated from rhizomes of *Polygonum amplexicaule* D. Don var. sinense Forb (PADF). We found that APA has anti-tumor effects in a breast cancer xenograft mouse model and induces apoptosis in breast cancer cell lines. APA increased levels of cleaved caspase-3,-8,-9 and PARP, which resulted from suppression of MCL-1 and BCL-2 expression in the cells. APA also inactivated the Akt/mTOR pathway in breast cancer cells. Thus, APA exerts a strong anti-tumor effect on breast cancer cells, most likely through induction of apoptosis. Our study is the first to identify this novel anti-tumor compound and provides a new strategy for isolation and separation of single compounds from herbs.

## INTRODUCTION

Breast cancer is the most commonly occurring malignancy and the second leading cause of cancer-related death among women world-wide [1]. In the U.S., there are about 230,000 new cases each year in women and about 2,300 new cases in men [1, 2]. In China, approximately 30 in every 100,000 women will develop breast cancer in their lifetime, and this proportion is rising as the disease becomes more common in younger patients [3, 4]. Surgery is the first choice for breast cancer therapy; however, chemotherapy is still used as the main treatment for women whose cancer has spread outside the breast and axilla. All chemotherapy protocols currently in use have side effects. Therefore, new drugs with fewer side effects could have major impacts on breast cancer therapy.

Traditional Chinese Medicine (TCM) has become an important source of diverse therapeutic agents [5]. Natural products can serve as the origins of novel antitumor and immune stimulating reagents [6, 7]. TCM therapies are often multiple components, multiple targets and multiple activities, like the combination chemotherapies used in cancer treatment [6–8]. For example, flavonoids exhibit antioxidant, anti-inflammatory and anti-cancer properties [9–12] in several human cancers [13, 14]. The tumor suppressive effects of flavonoids are mediated through the induction of apoptosis and cell cycle arrest that results from interference with key intracellular signaling pathways [15, 16]. The rhizomes of *Polygonum amplexicaule* D. Don var. sinense Forb (PADF) are considered to be a restorative food in some areas of China. Earlier studies demonstrated that PADF has anti-tumor activity [6], and we previously found that the primary anti-tumor components of PADF were the total flavonoids [7]; however, it is unclear if and how PADF effects breast cancer.

Here we used a new protocol to isolate the polar compounds amplexicaule A (APA) and B (APB) from the n-butanol fraction of PADF. APA and APB are flavonoid glucosides first isolated from *Comastoma pedunculatum* and *Clausena lansium*, respectively [17, 18], but up to now there have been no reports on their biological activities. In this study, we show that APA induces apoptosis, which likely contributes to the anti-cancer actions of PADF. The apoptosis-inducing activity of APA is associated with the downregulation of MCL-1 and BCL-2 proteins and activation of caspases-3, -8 and -9. We also found that APA suppresses the phosphorylation of Akt, thereby inactivating the Akt/mTOR pathway and relaying apoptosis signaling. Our data demonstrate that APA extracted from PADF has anti-tumor effects on breast cancer via induction of apoptosis. Our findings suggest that APA may be a novel, natural treatment for breast cancer.

## RESULTS

### The molecular structure of the two compounds

**Figure 1:**
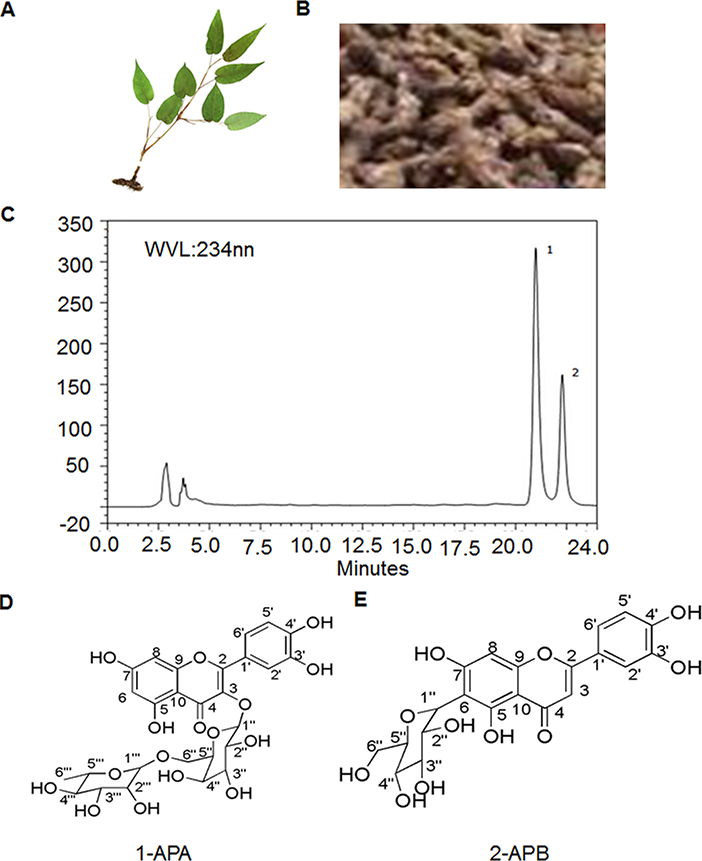
Extraction and purification of APA, APB in PADF (**A**) Photo of PADF; (**B**) Root of PADF used for isolation; (**C**) Two single compounds identified in extracts of PADF; (**D**) Molecular structure of APA; (**E**) Molecular structure of APB.

PADF (Figure 1A) is a plant growing in the area of Enshi, Hubei province, China. The root part (Figure 1B) of the plant was prepared for isolation of total flavonoids. The detail protocol for isolation and separation is described in the methods. Two peaks showed in the Fraction Fe of the total flavonoids from PADF on High Performance Liquid Chromatography (HPLC) flow (Figure 1C). The two compounds (compound-1 and compound-2) were further isolated through semi-prepared HPLC separation. They were confirmed as two distinctive compounds by spectroscopic methods, including: infrared (IR), ultraviolet (UV), mass spectroscopy (MS), proton nuclear magnetic resonance (^1^H-NMR), carbon-13 nuclear magnetic resonance (^13^C-NMR) and heteronuclear multiple-bond correlation spectroscopy (HMBC). The two compounds were named Amplexicaule A (APA) and Amplexicaule B (APB), respectively. Their molecular structures are identified as 3-o-[α-L-rhamnopyranosyl-(1-6)- β-D-Galactopyranose]-5,7,3′,4′-tetrahydroxy flavonoid (Figure 1D) [19] and 6-C-glucose-5,7,3′,4′-tetrahydroxy flavonoid (Figure 1E) [20] with molecular formulas of C_27_H_30_O_16_ and C_21_H_20_O_11_, respectively.

### The *in vivo* anti-tumor effects of APA in a breast cancer xenograft mouse model

**Figure 2:**
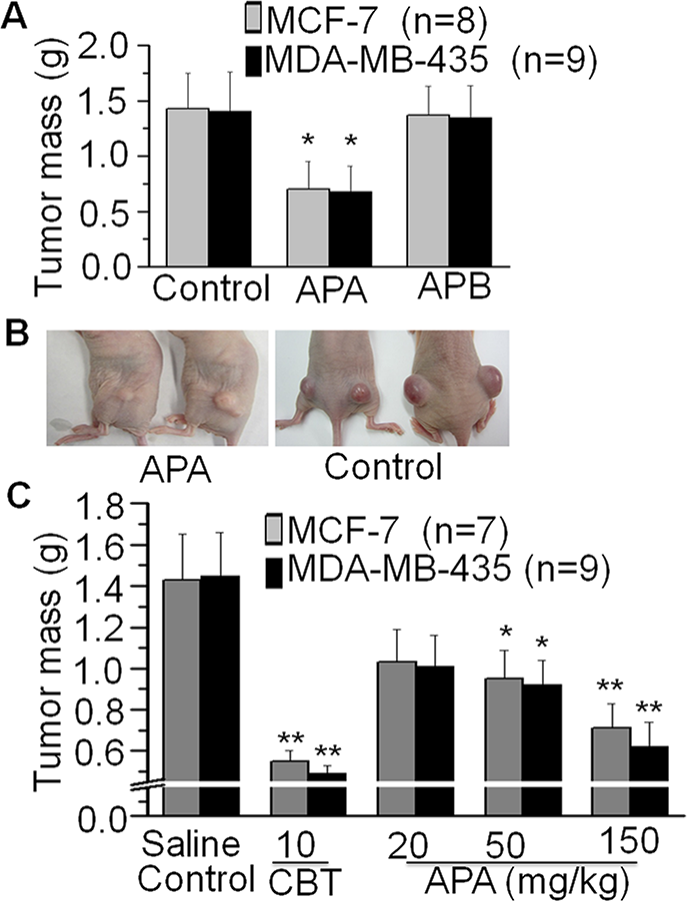
Suppressive effects of APA on tumor growthin a xenograft breast cancer mouse model (**A**) Effects of APA or APB on tumor mass in a breast tumor cells xenograft mouse model; (**B**) Comparison of a representative engrafted tumor on nude mice between the APA treatment group and control group; (**C**) APA alters tumor mass in a dose-dependent manner. *p < 0.05; **p < 0.01.

In order to explore the anti-tumor effects of APA and APB, we first tested their effects *in vivo* with MCF-7 or MDA-MB-435 xenograft mouse models. As shown in Figure 2A and Supplementary Tables 1 and 2, APA had an inhibitory effect on tumor mass in both the MCF-7 and MDA-MB-435 xenograft models (*p* < 0.01), compared to saline treatment. In contrast, APB had no tumor-suppressive activities *in vivo*. We further tested APA at various doses using Capecitabine Tablets, a conventional anti-breast cancer drug, as a positive control. Tumor masses in the 50 and 150 mg/kg groups of APA treatment were less than that of non-treatment groups (*p* < 0.05 or *P* < 0.01) without obvious body weight changes in the mice (Figure 2B, 2C and Supplementary Tables 3 and 4), indicating that APA suppresses tumor growth in a dose-dependent manner. Although the Capecitabine Tablets had a higher tumor inhibitory rate, the average body weight of the Capecitabine treated mice was also decreased (*p* < 0.05) compared to saline treatment (Supplementary Table 3). In addition to body weight (Supplementary Tables 1–4) we also examined serum indicators of hepatic and renal functions (Supplementary Figure 5 and 6), and blood counts in these mice (Supplementary Table 7), and found no differences between APA treatment groups and controls.

### APA inhibits proliferation of human breast cancer cells

**Figure 3:**
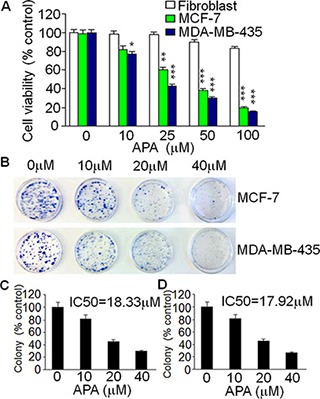
Suppressive effect of APA on cell growth of breast cancer cells *in vitro* (**A**) Cytotoxicity of APA specifically on breast cancer MCF-7 and MDA-MB-435 cells in a dose-dependent manner; (**B**) Effect of APA on colony numbers of MCF-7 and MDA-MB-435 by a colony forming assay; (**C**–**D**) Quantitation of the colony forming assay for MCF-7 (C) and MDA-MB-435 (D) cells.*p < 0.05; **p < 0.01; ***p < 0.001.

To understand the mechanism underlying APA's anti-tumor actions, we examined the effects of APA on cell proliferation. Human breast cancer cell lines MCF-7 and MDA-MB-435 and human fibroblasts were treated with various concentrations of APA. After treatment, cell viability was examined using a MTT assay. Treatment with APA inhibited tumor cell viability in a dose-dependent manner while having very little effect on the proliferation of fibroblast cells (Figure 3A). These results suggest that APA specifically inhibits the proliferation of tumor cells but not normal cells. To further demonstrate the antiproliferative activity of APA, we carried out a clonogenic assay. APA inhibited the clonogenicity of MCF-7 and MDA-MB-435 cells in a dose-dependent manner (Figure 3B). About 70% inhibition of colony formation was observed at 40 *μ*M of APA (Figure 3C and 3D).

### Apoptogenic effects of APA on human breast cancer cells

**Figure 4:**
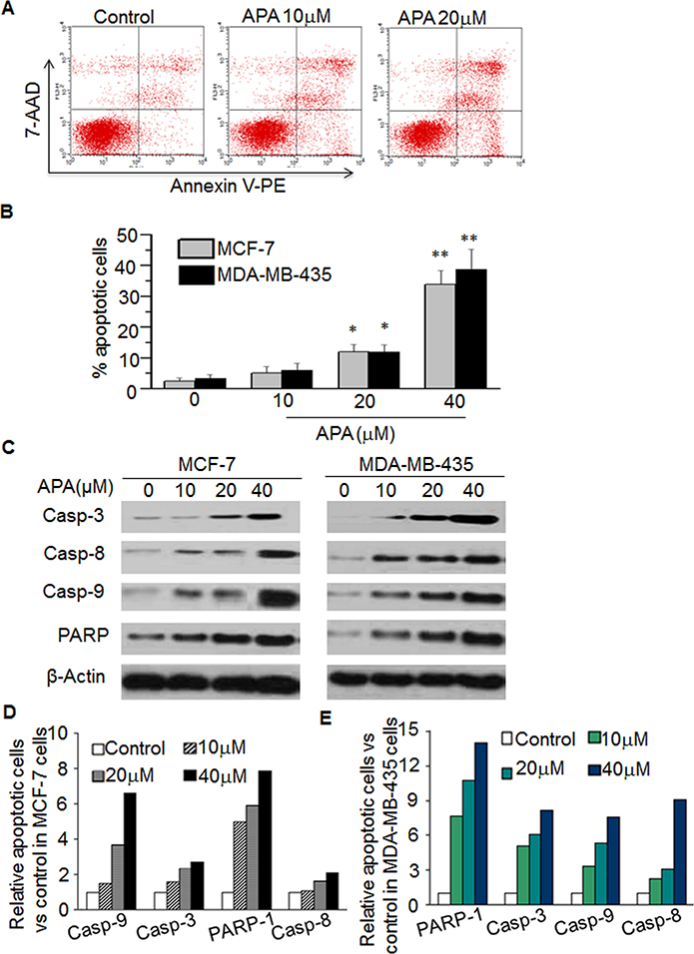
APA induced apoptosis by increasing expression of Casp-3,-8,-9 and PARPin MCF-7 and MDA-MB-435 cells (**A**–**B**) Effect of APA on apoptosis of MCF-7 cells. Cells were stained with Annexin V-PE and the apoptotic cells were analyzed with flow cytometry. (A) is the representative data in MCF-7 cells and (B) is quantitation data for MCF-7 cells and MDA-MB-435 cells; (**C**) Effect of APA on cleaved Casp3,-8,-9 and PARP by western blot; (**D**–**E**) Quantitation of the western data for MCF-7 (D) and MDA-MB-435 (E) cells. *p < 0.05; **P < 0.01.

To determine whether the antiproliferative effects of APA is associated with the induction of apoptosis, tumor cells treated with APA were sorted via flow cytometry following Annexin V/7-AAD staining (a marker of apoptosis). Figure 4A shows the representative flow images in MLF-7 cells. APA increased the percentage of apoptotic cells in both MCF-7 and MDA-MB-435 lines in a dose-dependent manner as compared with vehicle treatment (Figure 4B). As caspases are important regulators of apoptosis [21], to further confirm APA-induced apoptosis, we assessed the effects of APA on caspase activation. Treatment of tumor cells with APA resulted in an elevation of the cleaved form of PARP, as well as caspases-3, -8, and -9 in a dose-dependent manner (Figure 4C), indicating that APA may induce apoptosis through activation of these molecules.

### APA decreases MCL-1 and BCL-2

**Figure 5:**
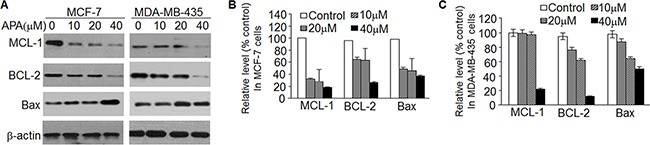
APA suppressed the expression of anti-apoptotic modulators (**A**) Expression of MCL-1, BCL-2 and Bax in the indicated doses of APA-treated MCF-7 (left panel) and MDA-MB-435 (right panel) cells; (**B**–**C**) Relative expression of MCL-1, BCL-2, Bax and β-actin vs non-treatment control for panel A in MCF-7 (B) and MDA-MB-435 (C) cells.

**Figure 6:**
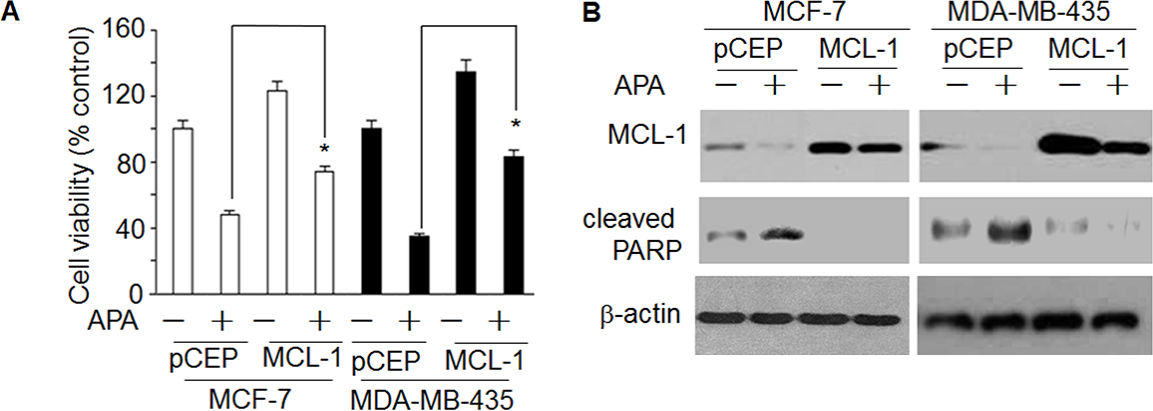
Over-expression of MCL-1blocked the effect of APAon decreasedcleaved PARP and increased cell death (**A**) Effect of enhanced expression of MCL-1on APA-induced increase of cell death; (**B**) Effect of enhanced expression of MCL-1on APA-induced decrease of cleaved PARP. *p < 0.05.

To further understand how APA induces apoptosis, we measured the expression of the BCL-2 family proteins, a group of proteins that regulate the anti- and pro-apoptotic processes [22]. APA treatment down-regulated MCL-1 and BCL-2 proteins in both MCF-7 and MDA-MB-435 cells (Figure 5A). In addition, there was a slight increase in Bax expression following APA treatment (Figure 5A). As MCL-1 was down-regulated in the cells treated with APA, we next sought to demonstrate the functional significance of MCL-1 in APA-induced apoptosis. We transfected MCF-7 and MDA-MB-231 cells with a MCL-1 expression vector and then tested the effects of APA on tumor cell viability and apoptosis. Cells transfected with an empty vector were used as controls. Expression of MCL-1 blocked APA-induced cytotoxic effects on MCF-7 and MDA-MB-435 cells and blocked apoptosis, as evidenced by the near absence of cleaved PARP in MCL-1 over-expressing cells (Figure 6B). These results suggest that downregulation of MCL-1 is necessary for APA-induced apoptosis in breast tumor cells.

### APA inactivates the Akt/mTOR signaling pathway

**Figure 7:**
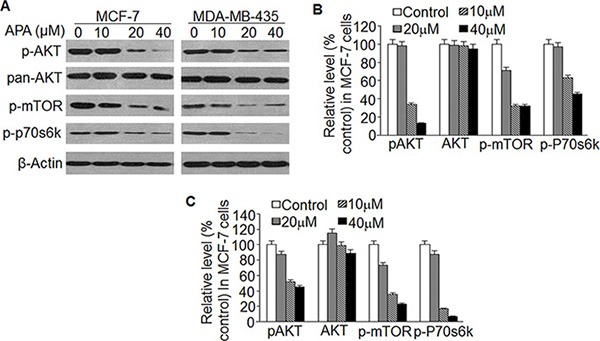
Effect of APA on suppression of the AKT/mTOR pathway (**A**) Effect of APA on phosphorylation of AKT, mTOR and p70s6k; (**B**–**C**) Relative level of p-AKT, p-mTOR and p-p70s6k vs non-treatment control in MCF-7 (B) and MDA-MB-435 (C) cells.

Flavonoid and its metabolites act upon the Akt/mTOR pathway. In order to understand the upstream signals responsible for APA-induced apoptosis, we examined the effect of this compound on Akt/mTOR signaling. We found that treatment with APA decreased levels of phospho-Akt, phospho-mTOR, and phospho-P70 S6 kinase, but did not alter total Akt protein (Figure 7A). Our results indicate that APA suppresses the activation of Akt/mTOR pathways.

### APA induces cytotoxicity in primary breast tumor cells

**Figure 8:**
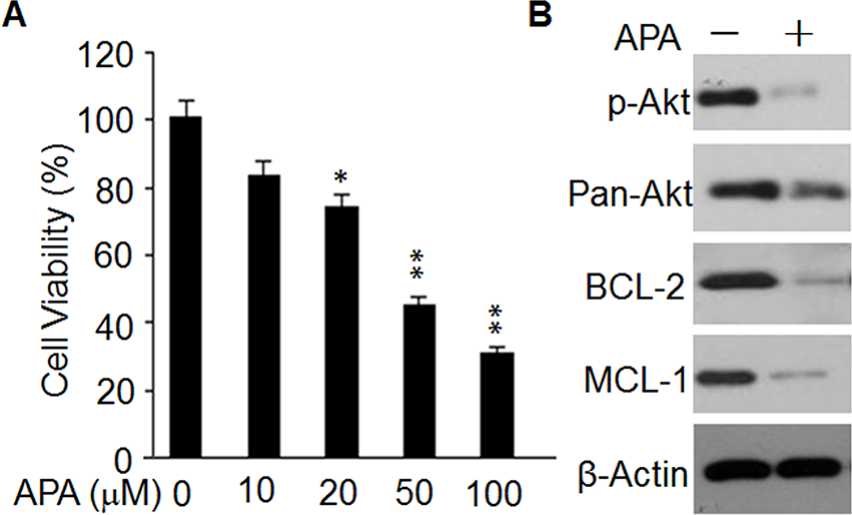
Effect of APA on cell proliferation, p-AKT level, and expression of BCL-2 and MCL-1in primary breast cancer cells (**A**) Cytotoxicity of APA specifically on primary breast cancer cells in a dose-dependent manner; *p < 0.05; **p < 0.01. (**B**) Effect of APA on p-AKT, BCL-1 and MCL-1 in primary breast cancer cells; the level of p-AKT, BCL-1 and MCL-1 was detected by western blot.

Finally, we assessed the effects of APA on cytotoxicity of primary tumor cells from breast cancer patients. After 2 days of APA treatment the number of viable cells was reduced in a dose-dependent manner (Figure 8A). APA treatment at a dose of 100 ng/mL also reduced p-AKT, MCL-1, and BCL-2 expression in these cells (Figure 8B). These data demonstrate a cytotoxic effect of APA on primary breast cancer cells acting through the same mechanism as in breast cell lines.

## DISCUSSION

Flavonoids are the main components responsible for anti-tumor activity in plant extracts. The method we used to extract total flavonoids is well established, as was described by Wallace and Burong [23, 24]. We previously isolated total flavonoids from PADF using basic water extraction followed by acidic precipitation, and reported that the extracts had anti-tumor effects [7]. We have now used a new strategy to further isolate single compounds from PADF, structurally identified the isolated compounds and screened them for their anti-tumor activities. Using this approach we found that a single compound is responsible for the anti-tumor effects of PADF.

In the isolation process, we first extracted the PADF with 95% ethanol, after which the total extract was further separated based on the polarity of the compounds into petroleum ether, ethylacetate, n-butanol and aqueous fractions. We tested the anti-tumor effects of the extracted fractions and found that the n-butanol fraction had strong anti-tumor effects against breast cancer cells. The n-butanol fraction was then further separated using semi-prepared HPLC, and two single compounds (APA and APB) were obtained. We identified their molecular structures, and found that both are polar flavonoid glycosides. We further found that APA but not APB exerts anti-tumor effects by inducing apoptosis, which may be due to APA has stronger polarity. We also examined the molecular mechanisms underlying APA-induced the apoptosis and found that APA induces apoptosis by inactivating Akt, suppressing MCL-1 and BCL-2 expression, and activating capases-3, -8, and -9 (for a model, See Supplementary Figure 1). Our study not only identifies the single compound in PADF responsible for anti-tumor effects, but also provides a new strategy and protocol for isolation of single compounds with anti-tumor activities from herbs.

Plant-derived chemicals are drawing increasing attention as sources of therapeutic reagents, in part due to their lower toxicity toward the host [7, 25]. In our study, the weights of transplanted tumors collected from the APA-treated group were smaller than those from the saline control group. APA treatment suppressed breast cancer cell proliferation and induced apoptosis in a dose-dependent manner. These data clearly demonstrate the *in vivo* tumor suppressing activity and *in vitro* apoptosis-inducing activity of APA against breast cancer cells. Importantly, APA had no effect on normal fibroblasts *in vitro*, nor was there an effect on body weight in the xenograft mouse model. In addition, the parameters of routine blood examination and indicators of hepatic and renal function were all within the normal range after APA treatment in the mouse model. Taken together, these observations suggest that APA, at the dosages used in our study, exhibited few side effects on host animals. This finding is also consistent with our earlier report on the effects of total PADF extract, which was shown to be nontoxic [7].

Many anti-tumor chemicals exert their antiproliferative effects by inducing apoptosis [26, 27]. In this study, we also observed that APA inhibited tumor cell proliferation by inducing apoptosis. Apoptosis results from the activation of cascade of caspases, a family of proteases comprising the central component of the proteolytic system in the apoptotic process [21]. We found that APA induced cleavage of caspases-3, -8 and -9 and also resulted in PARP cleavage, another apoptosis-related event, possibly catalyzed by caspase-3. These results indicate that APA exerts its anti-tumor effects by inducing apoptosis in breast cancer cells.

The mitochondrial apoptosis pathway is an important means of inducing cell death, and flavonoids have been reported to activate mitochondrial apoptotic signaling [14, 28, 29]. As critical inhibitors of the mitochondrial apoptotic pathway [30], BCL-2 family proteins bind to the outer membrane of mitochondria and prevent release of cytochrome c [31]. APA treatment reduced BCL-2 levels in MCF-7 and MDA-MB-435 cells, suggesting downregulation of BCL-2 family proteins contribute to the promotion of apoptosis by APA. Notably, APA treatment also led to downregulation of MCL-1, a pro-survival member of the BCL-2 family [32]. These results suggest that APA-induced downregulation of MCL-1 plays an essential role in the APA-induced tumor cell apoptosis.

The Akt/mTOR pathway is important for the regulation of cell proliferation, growth and apoptosis [33, 34]. Flavonoid and its metabolites interact with Akt/mTOR, affecting its function [35, 36]. Molecular similarity analysis showed that APA strongly affects Akt activation (data not shown). Moreover, our data demonstrate that APA inhibits Akt/mTOR and P70 S6k in breast cancer cells. Flavonoids-induced Akt/mTOR inactivation is also reportedly responsible for suppression of BCL-2 and MCL-1 [14, 37]. We hypothesize that APA-induced inactivation of Akt/mTOR pathways is responsible for the downregulation of BCL-2 and MCL-1 in the treated cells, which triggers apoptosis.

Finally, we observed that APA exerts antiproliferative effects through inactivation of Akt and suppression of BCL-2 and MCL-1 in primary cancer cells from breast cancer patients. These data further support the notion that APA could potentially be an effective therapeutic agent for the treatment of breast cancer.

## MATERIALS AND METHODS

### Extraction and purification of amplexicaule A, B

Rhizomes of Polygonum *amplexicaule* D. Don var. sinense Forb (PADF) were collected in Enshi, Hubei Province, PR China and identified by Dr. Dingrong Wan's laboratory, College of Pharmacy, South-Central University for Nationalities, China. Voucher samples (No. SC-2012187) were deposited at the Herbarium of Medical Plants located in the College of Pharmacy, South-Central University for Nationalities. The dried root tubers of PADF (10 kg dry weight for each lot) were extracted with 95% alcohol three times at room temperature. The combined solution was filtered and concentrated under reduced pressure to produce 95% ethanol extract. The EtOH extract was suspended with a solution of water:MeOH (1:9) and successively extracted with petroleum ether, ethylacetete, and n-butanol. The yield of 95% ethanol extract and extractive fractions were weighed and dried to constant weight, and kept in a desiccator. The respective yields were: ethanol extract fraction 1.185 kg, petroleum ether fraction 35.3 g, ethyl acetate fraction 490.0 g, n-butanol fraction 402 g, and residuary water fraction 258.0 g. The n-butanol fraction was repeatedly separated by normal phase silica gel column and eluted with a Chloroform: Methanol step gradient (1:9, 2:8, 3:7……9:1) to obtain 7 fractions (Fr A to Fr G). Fraction D was separated by Reverse-silica gel (YMC Co. Japan) column and eluted with methanol-water in the same step gradient to generate 7 fractions (Fr Fa to Fg). Fe (120 mg) was separated by semi-preparative HPLC. For HPLC, the mobile phase was acetonitrile:water (28:72) mixture and the semi-preparative column was Super Co. Inc. Waters S Spheripor ODS (particle size 5 μm, diameter 10 mm, length 250 mm). Flow rate was set at 1.5 ml/min, and injected 100 μL at a time; the detection was performed at 254 nm. The temperature of the column oven was set at 35°C (Figure 1). Two compounds (compound-1 15 mg and compound-2 10 mg) were obtained and named. They were identified by spectrum methods (including IR, UV, MS, ^1^H-NMR, ^13^C-NMR and HMBC).

### Patients' samples

Ten breast cancer tissue specimens were obtained from patients with breast cancer stage IV at Renmin Hospital of Wuhan University, PR, China. Informed consent was obtained from the patients and the study was approved by the ethics committee of Renmin hospital of Wuhan University, PR, China and ethics committee of South-Central University for Nationalities, Wuhan, PR China.

### Tumor cell preparation

Tumor cells were isolated from sterile tumor specimens (surgical biopsy, malignant effusion, or surgery tumor tissues) as previously reported [38, 39]. To be evaluable, each specimen had to achieve at least 90% pure tumor cell content morphologically [38, 39] and 90% viability by trypan blue exclusion. The resulting cells were cultured in a reported media [38] and treated with indicated dose of APA at a concentration of 2 × 10^5^ cells/ml for 2 days. Then cell viability was analyzed with MTT and cell lysates were prepared for western bot as described below.

### Reagents and antibodies

Amplexicaule A, B (purity = 99.5%) were dissolved in dimethyl sulfoxide (DMSO; Sigma, St. Louis, MO) at concentration of 250 mg/ml, and stored at −20°C. Polyclonal antibodies against MCL-1, BCL-2, BAX, cleaved-caspase-3, -8, -9, cleaved-PARP, phospho-Akt, Akt, phospho-mTOR, phospho-P70 S6k and β-actin were purchased from Cell Signaling (Beverly, MA). Horseradish peroxidase (HRP)-conjugated anti-rabbit immunoglobulin G (IgG) was purchased from Abcam Biotechnology (USA). All cell culture media and other reagents were purchased from Invitrogen (Carlsbad, CA). Western blot reagents were obtained from Pierce Biotechnology.

### Cells, animals and animal experimentation

MCF-7 and MDA-MB-435 cells were obtained from ATCC and cultured in Dulbecco's modified Eagle's medium (Gibco, Grand Island, NY) supplemented with 10% fetal bovine serum (Gibco, Grand Island, NY), 100 U/mL penicillin, and 100 *μ*g/mL streptomycin, and maintained in a humidified atmosphere of 5% CO_2_ at 37^°^C with 2 passages weekly. All cultures were monitored routinely and found to be free of contamination by mycoplasma or fungi. All lines were discarded after 3 months and new lines propagated from frozen stocks.

Female Kunming *nude mice* (18–22 g), aged 5–6 weeks, were purchased from the Experimental Animal Center, Institute of Health and Epidemic Prevention (Wuhan, China). Mice were housed in a standard specific pathogen free (SPF) environment. All the animal experimental procedures were approved by the Animal Care and Use Committee of South-Central University for Nationalities (Wuhan, China). To establish a transplanted tumor model, 1 × 10^7^ of MCF-7 cells (Viable cells > 95%) were injected subcutaneously into the back of the nude mice using a 22-gauge needle. Strict aseptic technique was followed. The mean volume of the implanted tumors at the time of inoculation was about 5 mm^3^, without differences among groups. The mice were randomly divided into several groups as described in the text, with 10 mice per group. Drug administration began 3 days later. The positive control group received Capecitabine Tablets (CBT) at a dosage of 10 mg/kg. The vehicle control group received 0.9% normal saline with DMSO. The drugs were administered by intraperitoneal injection for 10 days at different doses (mg/kg/day) as indicated in Figure 2A and 2C. All mice were then euthanized and the segregated tumors were weighed immediately.

### Cell viability assay

The cytotoxic effect of APA on tumor cells was determined using 3-(4, 5-dimethylthiazol-2-yl)-2, 5-diphenyltetrazoliumbromide (MTT) staining (28). Briefly, 5 × 10^3^ cells/well were plated in 96-well plates with 100 *μ*L of culture medium for 24 h and then exposed to different concentrations of APA. After 72 h, the culture medium was replaced with 100 *μ*L of fresh medium including 0.5 mg/mL MTT. Following 4 h of incubation 37°C, this medium was removed, and 100 *μ*L of DMSO was added to each well to dissolve the purple formazan crystals. The color reaction was quantified using an automatic plate reader (Bio-Tek Instrument Inc, Winooski, VT) at 570 nm. The half maximal inhibitory concentration (IC_50_) (in *μ*M), which represents the concentration of the drug that lowers the cell number by 50%, was calculated from the concentration-response curve. Each experiment was repeated three times (*n* = 3), and the average IC_50_ shown.

### Clonogenicity assay

This assay was carried out as previously reported (28). Briefly, 1 × 10^3^ cells/plate were plated in 3.5-mm flat-bottom plates with 2 mL of culture medium for 24 h and then exposed to different concentrations of APA. After 7 days of incubation in a humidified atmosphere of 5% CO_2_ at 37°C, the supernatant was discarded and the cells were washed with PBS before 1 mL crystal violet solution [0.5% (w/v) in methanol] was added in each plate. Plates were incubated under shaking for 10 min at room temperature. After washing 3 times with tap water, the plates were air dried at room temperature. Colonies were photographed and counted using ImageJ imaging software (NIH). Three independent experiments were performed, and each experiment was carried out in triplicate.

### Apoptosis assay

APA-induced apoptosis in MCF-7 and MDB-MA-435 cells was determined by flow cytometry using the Annexin V-PE Apoptosis Detection Kit following the manufacturer's instructions (BD Bioscience) (28–29). Briefly, 3 × 10^5^ cells were treated with APA (0, 10, 20, 40 μM) for 48 h. The cells were then harvested, washed in PBS, and incubated with Annexin V and 7-AAD for staining in binding buffer at room temperature for 10 min in the dark. The stained cells were analyzed using the BD FACSCalibur.

### Western blot analysis

Cells were lysed in the M-PER mammalian protein extraction reagent (Thermo Scientific) supplemented with a protease inhibitor cocktail (Roche, Indianapolis, IN) at room temperature for 5 min, followed by centrifugation at 14,000 × g for 10 min. Protein concentrations of cell lysates were measured using the Bio-Rad DC assay reagent (Bio-Rad, Hercules, CA). Proteins (10–20 *μ*g) were resolved by SDS-PAGE and then transferred to polyvinylidene difluoride (PVDF) membrane (Bio-Rad, Hercules, CA). The PVDF membranes were incubated with the respective antibodies in 3% BSA/TBST at 4°C overnight, followed by incubation with secondary antibody at room temperature for 1 h. Protein signals were detected by enhanced chemiluminescence method, according to the manufacturer's protocol. Statistical analysis

All presented data and results were confirmed by at least 3 independent experiments. The data are expressed as the means ± SD. Statistical analysis was performed using the Student's *t*-test, with the following significance levels: *P < 0.05 and **P < 0.01.

## SUPPLEMENTARY MATERIALS TABLES


