# Supplementary material for: Amplexicaule A exerts anti-tumor effects by inducing apoptosis in human breast cancer
Source: Oncotarget. 2016 Mar 2;7(14):18521–30. doi: 10.18632/oncotarget.7848 (PMC4951306; doi:10.18632/oncotarget.7848)
Supplement: Supplementary file 1 [file oncotarget-07-18521-s001.pdf]

Amplexicaule A exerts anti-tumor effects by inducing apoptosis in human breast cancer

Supplementary Materials

Supplementary Table S1: Effect of APA and APB from PADFon xenografted tumor growth in MCF-7 ells

Group	Dose	Mice weight (g)		Tumor weight
	mg/kg	Start	End	(g)
Saline	–	20.5 ± 1.2	27.3 ± 1.1	1.43 ± 0.32
APA	150	21.2 ± 1.3	27.4 ± 0.9	0.70 ± 0.25*
APB	150	21.1 ± 1.1	27.1 ± 1.2	1.37 ± 0.26

*$P < 0.05$, compared to tumor-bearing mice treated with saline.

Supplementary Table S2: Effect of APA and APB from PADF on xenografted tumor growth in MDA-MB-435 cells

Group	Dose	Mice weight (g)		Tumor weight
	mg/kg	Start	End	(g)
Saline	–	20.2 ± 1.3	27.1 ± 1.0	1.41 ± 0.35
APA	150	20.8 ± 1.5	27.1 ± 0.9	0.68 ± 0.23*
APB	150	21.4 ± 1.5	27.3 ± 1.1	1.35 ± 0.29

*$P < 0.05$, compared to tumor-bearing mice treated with saline.

Supplementary Table S3: Effect of APA from PADF on xenografted tumor growth of MLF-7 cells

Group	Dose	Mice weight (g)		tumor
	mg/kg	Start	End	(g)
Non-tumor	–	21.6 + 0.7	26.8 + 0.8	–
Saline	–	21.8 + 0.4	27.0 + 0.7	1.43 + 0.22
CBT	10	21.7 + 0.6	26.1 + 1.0	0.55 + 0.05**
APA	20	22.1 + 0.5	27.4 + 1.1	1.03 + 0.16
APA	50	21.9 + 0.4	27.1 + 1.2	0.95 + 0.14*
APA	150	21.8 + 0.6	27.9 + 1.1	0.71 + 0.12**

*$P < 0.05$, compared to tumor-bearing mice treated with saline.

Supplementary Table S4: Effect of APA from PADF on xenografted tumor growth of MDA-MB-435

Group	Dose	Mice	weight (g)	tumor weight
	mg/kg	Start	End	(g)
Non-tumor	−	21.4 + 0.6	26.9 + 0.6	−
Saline	−	20.8 + 0.4	27.1 + 0.7	1.41 + 0.21
CBT	10	21.5 + 0.5	26.1 + 1.0	0.49 + 0.04**
APA	20	21.1 + 0.5	27.2 + 1.3	1.01 + 0.15
APA	50	21.4 + 0.2	27.3 + 1.1	0.92 + 0.12 *
APA	150	21.5 + 0.5	27.1 + 1.4	0.62 + 0.12 **

*$P < 0.05$, compared to tumor-bearing mice treated with saline.

Supplementary Table S5: Effect of APAon serum indexes of liver function

Group	Dosage (mg/kg)	ALT (1U/L)	AST (1U/L)
Non-tumor	−	118.9 ± 12.7	512.4 ± 30.6
Saline	−	117.9 ± 12.5	510.6 ± 23.1
CBT	10	293.1 ± 16.8*	832.7 ± 24.9*
APA	20	116.6 ± 10.9	515.7 ± 24.1
APA	50	120.9 ± 11.5	513.8 ± 20.9
APA	100	118.4 ± 12.9	518.7 ± 25.9
APA	150	119.4 ± 11.8	519.4 ± 24.7

*$P < 0.05$, compared to tumor-bearing mice treated with saline.

Supplementary Table S6: Effect of APAon renal function indexes in tumor-bearing mice

Group	Dosage (mg/kg)	BUN (1U/L)	UA(1U/L)	CRE(1U/L)
Non-tumor	−	6.88 ± 0.49	132.8 ± 9.7	46.10 ± 1.43
Saline	−	6.98 ± 0.45	133.6 ± 9.1	46 ± 1.78
CBT	10	11.98 ± 0.89*	138.1 ± 8.9	64.78 ± 2.14*
APA	20	6.87 ± 0.61	134.4 ± 8.6	45.10 ± 1.28
APA	50	6.79 ± 0.51	130.5 ± 9.2	46 ± 1.87
APA	100	6.84 ± 1.61	132.7 ± 9.4	46.32 ± 1.79
APA	150	6.86 ± 1.54	131.9 ± 9.6	45.89 ± 1.68

*$P < 0.05$, compared to tumor-bearing mice treated with saline.

Supplementary Table S7: Effect of APAon WBC, RBC, HGB and PLT in tumor-bearing mice

Group	Dose	WBC (10^9/L)	RBC (10^{12}/L)	HGB (g/L)	PLT (10^9/L)
Non-tumor		6.12 ± 0.49	712 ± 0.23	97.8 ± 4.1	685.9 ± 41.2
Saline		7.19 ± 0.62	7.36 ± 0.31	98.5 ± 4.2	701.9 ± 60.2
CBT	10	2.28 ± 0.44*	7.27 ± 0.24	96.5 ± 3.7	695.9 ± 41.8
APA	20	7.99 ± 0.64	7.41 ± 0.15	98.4 ± 3.9	686.1 ± 48.9
APA	50	7.67 ± 0.63	7.01 ± 0.16	97.3 ± 4.4	671.1 ± 55.4
APA	100	7.25 ± 0.44	7.38 ± 0.18	98.7 ± 3.9	695.8 ± 53.2
APA	150	7.31 ± 0.39	7.41 ± 0.22	99.4 ± 4.1	699.1 ± 50.2

*$P < 0.05$, compared to tumor-bearing mice treated with saline.
